# Seroprevalence of *Toxoplasma gondii* in horses and donkeys in Yunnan Province, Southwestern China

**DOI:** 10.1186/1756-3305-6-168

**Published:** 2013-06-06

**Authors:** Qiang Miao, Xi Wang, Li-Na She, Ya-Ting Fan, Fei-Zhou Yuan, Jian-Fa Yang, Xing-Quan Zhu, Feng-Cai Zou

**Affiliations:** 1 College of Animal Science and Technology, Yunnan Agricultural University, Yunnan Province 650201, Kunming, PR China; 2 Yunnan Academy of Scientific and Technical Information, Yunnan Province 650091, Kunming, PR China; 3 School of Life Sciences, Yunnan University, Yunnan Province 650091, Kunming, PR China

## Abstract

**Background:**

*Toxoplasma gondii* is an intracellular protozoan parasite that infects almost all warm-blooded animals, including humans, with a worldwide distribution. There have been limited reports about the seroprevalence of *T*. *gondii* infection in equids around the world and little is known about the seroprevalence of *T*. *gondii* in equids in southwestern China, in particular in Yunnan Province. The objective of the present investigation was to estimate the seroprevalence of *T*. *gondii* infection in equids in this area.

**Methods:**

A total of 399 serum samples (266 from horses and 133 from donkeys) were collected in 2012, and assayed for *T*. *gondii* antibodies by Indirect Haemagglutination (IHA) test using a commercially available kit.

**Results:**

A total of 108 (27.1%) equids, including 81 (30.5%) horses and 27 (20.3%) donkeys were positive for *T*. *gondii* antibodies, and the seroprevalence ranged from 18.8% to 37.5% among different sampling areas. The seroprevalence was 27.4% and 26.8% for male and female equids, respectively, and the difference was not statistically significant (*P* > 0.05). The seroprevalence ranged from 21% to 32.9% among different age groups, and the difference was not statistically significant (*P* > 0.05).

**Conclusions:**

The results of the present survey indicated the existence of high *T*. *gondii* seroprevalence in Yunnan Province, southwestern China, which has significant public health concern. Therefore, it is imperative that improved integrated measures be carried out to prevent and control *T*. *gondii* infection in equids in the studied region.

## Background

*Toxoplasma gondii* is an important zoonotic parasite, which can infect humans and almost all warm-blood animals, with a worldwide distribution [1-5]. Toxoplasmosis is not only of great importance for livestock and causes huge economic loss to the livestock industry, it is also a public health problem owing to its transmission to humans by ingestion of uncooked meat containing tissue cysts, or consuming food or drink contaminated with oocysts, or accidental ingestion of sporulated oocysts from the environment [1-5]. Although *T*. *gondii* infection rarely displays obvious clinical symptoms in adults, it may lead to severe consequences in an immunodeficient person such as an AIDS patient, and infection in pregnant women may lead to abortion, still birth, or other serious congenital consequences in newborns [1-3].

Horses and donkeys are important and useful animals to humans in many ways, such as sport competitions, police work, carriage, and so on. Horse and donkey meat are also the popular and delicate food for people worldwide. Infection of *T*. *gondii* is subclinical in horses, atypical clinical signs includes fever, ataxia, retinal degeneration and encephalomyelitis, and abortion or stillbirth in pregnant equids. Human toxoplasmosis cases associated with consumption of horse meat have been reported in some countries [6,7].

Yunnan Horse and Yunnan Donkey are fine equine breeds in China, they play very important roles in agriculture and tourism, and meat of horse and donkey are considered as special delicacy dishes in local restaurants in Yunnan Province. However, data on horse and donkey infection with *T*. *gondii* is limited in southwestern China. The objective of the present survey was to examine the seroprevalence of *T*. *gondii* infection in horses and donkeys in Yunnan Province, southwestern China, and the results would provide fundamental data for prevention and control of *T*. *gondii* infection in equine animals, and also will provide base-line information on potential risk factors associated with public health.

## Methods

### Ethics statement

The collection of serum samples from equids in the present study was consented by owners of equids, and all horses and donkeys were handled in strict accordance with good animal practice according to the Animal Ethics Procedures and Guidelines of the People's Republic of China.

### Sampled regions

**Figure 1:**
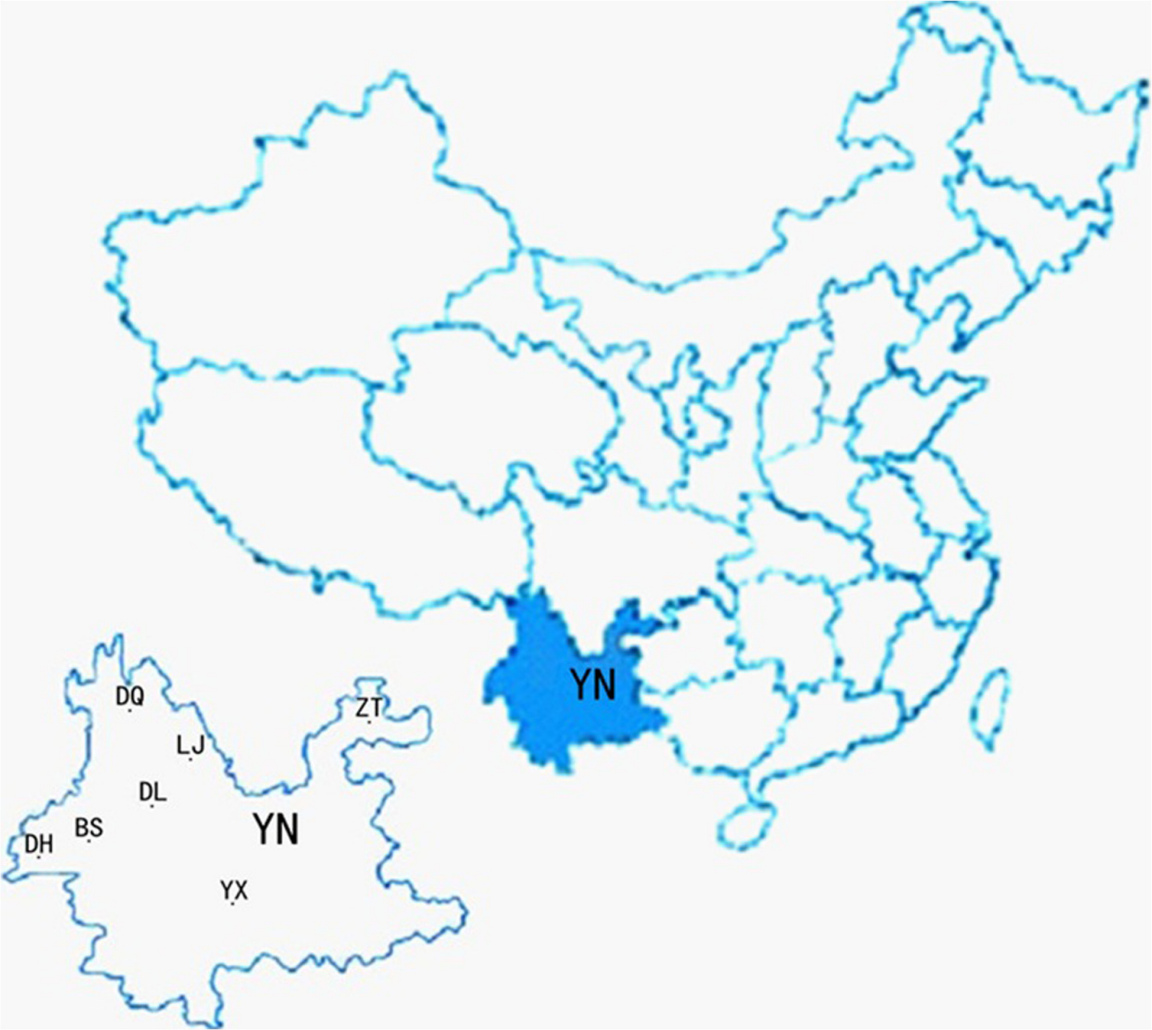
**Map showing Yunnan Province (YN), shadowed in southwestern China, where horse and donkey serum samples were collected for detection of *Toxoplasma gondii *antibodies.** The specific locations of sampling areas were marked in the enlarged map of Yunnan Province (YN), including Diqing state (DQ), Lijiang City (LJ), Dali state (DL), Baoshan city (BS), Dehong state (DH), Yuxi City (YX) and Zhaotong City (ZT).

The serum samples were collected from Diqing, Lijiang, Dehong, Baoshan, Yuxi and Zhaotong prefectures and municipalities of Yunnan Province, southwestern China (Figure 1).

### Blood samples

A total of 399 blood samples (266 from horses and 133 from donkeys) were collected via jugular vein of animals in 2012 in Yunnan Province. Whenever possible, data regarding species/breed, geographic origin, age and gender of each animal were collected. The ages of animals were classified into four categories according to their growth cycle: foal (0 < yr ≤ 1, 73 samples), adolescent (1 < yr ≤ 5, 108 samples), middle age (5 < yr ≤ 10, 138 samples) and elderly age (yr > 10, 80 samples), 190 were males and 209 were females. All samples were sent to the laboratory in Kunming and centrifuged (3,000 rpm) for 5 min, and the serum samples were kept at −20°C until assayed for antibodies to *T*. *gondii*.

### Serological assay

Antibodies to *T*. *gondii* were detected in serum samples by an indirect hemagglutination antibody (IHA) test using a commercially available kit (Veterinary Research Institute, Jiangsu Academy of Agricultural Sciences, Nanjing, China) according to the recommended protocol of the manufacturer for the detection of antibodies to *T*. *gondii* in animals. The serum sample was considered as positive when a layer of agglutinated erythrocytes was observed in wells with dilutions of 1:64 or higher.

### Statistical analysis

Statistical analysis of *T*. *gondii* seroprevalence in different regions, ages and genders of animals were performed using Generalized Lineal Model (GLM) test in the SPSS software (Release 18.0 standard version, SPSS Inc., Chicago, Illinois), and *P* < 0.05 was considered statistically significant.

## Results and discussion

108 of 399 examined equids were seropositive for *T*. *gondii* antibodies, the overall seroprevalence was 27.1%, and the seroprevalence ranged from 18.8% in Dehong to 37.5% in Diqing. 81 (30.5%) of the examined horses and 27 (20.3%) of the examined donkeys were seropositive, but the difference was not statistically significant (*P* > 0.05). The antibody titers were 1:64 in 38 horses and 13 donkeys, 1:128 in 20 horses and 5 donkeys, 1:256 in 13 horses and 2 donkeys, 1:512 in 2 horses and 1 donkey, 1:1024 in 8 horses and 6 donkeys. Seroprevalence in male and female equids was 27.4% and 26.8%, respectively, and the difference was not statistically significant (*P* > 0.05). Seroprevalence in animals of four different age groups were not significantly different (*P* > 0.05) (Table 1).

**Table 1:** **Seroprevalence of *Toxoplasma gondii *infection in equids in Yunnan province**, **southwestern China**

**Factor**	**Category**	**Sample size**	**Positive no.**	**Positive no. in different titers**				
			**(Seroprevalence %)**	**1:64**	**1:128**	**1:256**	**1:512**	**1:1024**
Species	Horse	266	81 (30.5)	38	20	13	2	8
	Donkey	133	27 (20.3)	13	5	2	1	6
Age	0 < yr ≤ 1	73	24 (32.9)	12	5	3	1	3
(year)	1 < yr ≤ 5	108	34 (31.5)	14	5	9	2	4
	5 < yr ≤ 10	138	29 (21)	12	11	2	0	4
	yr > 10	80	21 (26.3)	13	4	1	0	3
Gender	Male	190	52 (27.4)	25	12	6	1	8
	Female	209	56 (26.8)	26	13	9	2	6
Regions	Diqing	16	6 (37.5)	0	1	2	0	3
	Lijiang	83	23 (27.7)	16	7	0	0	0
	Dali	116	36 (31)	31	5	0	0	0
	Baoshan	39	9 (23.1)	2	5	2	0	0
	Dehong	16	3 (18.8)	1	1	1	0	0
	Yuxi	93	20 (21.5)	1	5	2	1	11
	Zhaotong	36	11 (30.6)	0	1	8	2	0
	Total	399	108 (27.1)	51	25	15	3	14

Under natural conditions, *T*. *gondii* seroprevalence in horses may vary from 0% to 90% [8]. In the present study, the overall average *Toxoplasma* seroprevalence was 27.1% in horses and donkeys in Yunnan Province, which was the highest among reported studies in China [9-20] (Table 2). *T*. *gondii* seroprevalence in horses was 30.5% in this survey, which was the second highest so far reported in the world. Previously reported *T*. *gondii* seroprevalence in horses were: 31.6% in Saudi Arabia by DT [21], 28.8% in Iran by MAT [22], 25% in Egypt by ELISA [23], 17.7% in Tunisa by MAT [24], 11.6% in Brazil by IFAT [25], 10.8% in Spain by MAT [26], 7.2% in Turkey by DT [27], 6.9% in North America by MAT [6], 6.1% in Mexico by MAT [28], 2.6% in South Korea by IFAT [29], and 1% in Sweden by DAT [30].

**Table 2:** **Seroprevalence of *Toxoplasma gondii *infection in equids in China**

**Species**	**Provinces**	**No. tested**	**Prevalence (%)**	**Serological test**	**Cut-off value**	**Time tested**	**References**
Horses	Liaoning	711	25	MAT^a^	1:25	2012	[9]
	Sichuan	242	4.55	IHA^b^	1:64	1982	[10]
	Liaoning	76	1.32	IHA	1:64	1989-1990	[11]
	Shangdong	147	0	IHA	1:64	1991-1993	[12]
	Gansu	149	2.7	IHA	1:64	unknown	[13]
	Qinghai	100	6	IHA	1:64	2000	[14]
	Xinjiang	60	0	IHA	1:64	unknown	[15]
	Hebei	43	2.33	IHA	1:64	unknown	[16]
Donkeys	Liaoning	738	23.6	MAT	1:25	2012	[9]
	Shangdong	17	5.88	IHA	1:64	1991-1993	[12]
	Xinjiang	30	0	IHA	1:64	unknown	[15]
	Hebei	33	26.06	IHA	1:64	unknown	[16]
Equids^*^	Ningxia	945	0.95	IHA	1:64	unknown	[17]
	Shanxi	1108	0.09	IHA	1:64	1986	[18]
	Henan	230	0.43	IHA	1:64	1988-1993	[19]
	Guangdong	149	2.7	IHA	1:64	1982	[20]

* Data for horses and donkeys were not separately reported in these references.

^a^ MAT: Modified Agglutination test.

^b^ IHA: Indirect Haemagglutination test.

The different seroprevalence results may due to differences in hygiene conditions, climates, and the prevalence of *T*. *gondii* in cats, as well as the sensitivity of the serological methods. Our preliminary survey showed that the prevalence of *T*. *gondii* oocysts in the faeces of stray cats in Yunnan was 25.6% (unpublished data), indicating a high risk as a source of *T*. *gondii* infection for equids, other animals and humans. In the present study, we used IHA to detect antibodies to *T*. *gondii* in horse and donkey serum samples because IHA is considered one of the most sensitive and specific serological methods for detecting *T*. *gondii* antibodies in equids, other animals and humans [31-35], and it have been extensively used in China (Table 2, [32-35]). The cutoff value of 1:64 was used according to the national standard (GB/T 18448.2-2008) of China for detection of *T*. *gondii* antibodies in humans and animals.

Due to the population size of donkeys in Yunnan Province, the sampled number (No = 133) of donkeys in the present study was small, and the results of the present survey may not reflect the accurate *T*. *gondii* seroprevalence in donkeys. Nevertheless, the present investigation revealed that horses and donkeys in Yunnan Province had a high *T*. *gondii* seroprevalence, indicating a potential threat to public health in this province, which is one of the most famous tourist destinations in China.

## Conclusions

The results of the present study indicate that *T*. *gondii* seroprevalence in horses and donkeys in Yunnan Province is quite high, consumption of horse or donkey meat is likely to be a risk factor for human infection with *T*. *gondii*. Therefore, it is imperative to take prevention and control measures to reduce *T*. *gondii* prevalence in equine animals in this province.

## Competing interests

The authors declare that they have no competing interests.

## Authors’ contributions

FCZ and XQZ conceived and designed the study, and critically revised the manuscript. QM, XW, and JFY performed the experiments, analyzed the data and drafted the manuscript, NLS, FZY and YTF helped in study design, study implementation and manuscript revision. All authors read and approved the final manuscript.

## References

[B1] Dubey JP Toxoplasmosis of Animals and Humans 2010 2 Boca Raton, Florida: CRC Press 313

[B2] Tenter AM Heckeroth AR Weiss LM *Toxoplasma gondii*: from animals to humans Int J Parasitol 2000 30 1217 1258 10.1016/S0020-7519(00)00124-7 11113252 PMC3109627

[B3] Zhou P Chen Z Li HL Zheng H He S Lin RQ Zhu XQ *Toxoplasma gondii* infection in humans in China Parasit Vectors 2011 4 165 10.1186/1756-3305-4-165 21864327 PMC3174123

[B4] Chen J Xu MJ Zhou DH Song HQ Wang CR Zhu XQ Canine and feline parasitic zoonoses in China Parasit Vectors 2012 5 152 10.1186/1756-3305-5-152 22839365 PMC3431282

[B5] Nardoni S Angelici MC Mugnaini L Mancianti F Prevalence of *Toxoplasma gondii* infection in *Myocastor coypus* in a protected Italian wetland Parasit Vectors 2011 4 240 10.1186/1756-3305-4-240 22196032 PMC3262763

[B6] Dubey JP Thulliez P Romand S Kwok OC Shen SK Gamble HR Serologic prevalence of *Toxoplasma gondii* in horses slaughtered for food in North America Vet Parasitol 1999 86 235 238 10.1016/S0304-4017(99)00148-X 10536980

[B7] Pomares C Ajzenberg D Bornard L Bernardin G Hasseine L Darde ML Marty P Toxoplasmosis and horse meat, France Emerg Infect Dis 2011 17 1327 1328 10.3201/eid1707.101642 21762609 PMC3381409

[B8] Tassi P *Toxoplasma gondii* infection in horses. A review Parassitologia 2007 49 7 15 18412038

[B9] Yang N Mu MY Yuan GM Zhang GX Li HK He JB Seroprevalence of *Toxoplasma gondii* in slaughtered horses and donkeys in Liaoning province, northeastern China Parasit Vectors 2013 6 140 10.1186/1756-3305-6-140 23680297 PMC3659062

[B10] Ling CW Wang PD Investigation report of *Toxoplasma gondii* antibodies to horse and mule in Sichuan region J Vet Sci Technol 1984 4 32 34 In Chinese

[B11] Ge LM Song LH Chen FY Wang M Yu XJ Yu YH Zhao QQ Lin RH Investigation of *Toxoplasma gondii* infection in humans and animals in Dalian City Chin J Zoonoses 1991 5 34 In Chinese

[B12] Fu B Wang ZM Epidemiological investigation of toxoplasmosis in Shandong Province Chin J Parasit Dis Cont 1995 8 205 207 In Chinese

[B13] Zhang DL Niu ZW Epidemiological investigation of toxoplasmosis in humans and livestock in Tianzhu region Gansu J Anim Vet Sci 1998 28 20 21 In Chinese

[B14] Gao LY Yang K Chen DT Seroprevalence of *Toxoplasma gondii* infection in horse in Tianjun region Chin J Anim Quarantine 2001 18 38 In Chinese

[B15] Bai WS Chen Y Ba LT Mai MT Investigation of toxoplasmosis in livestock in Aksu district usig serological method Chin J Vet Parasitol 2002 2 29 30 In Chinese

[B16] Cui P Fang SF Wu ZY Wu BQ Epidemiological survey of toxoplasmosis in livestock in Hebei Province Chin J Vet Sci Technol 2004 34 33 34 In Chinese

[B17] Zhang QQ Xu WP Wang XQ Chen YM Liu W Ma JW Li GD Du CB Wang Z Seroepidemiological survey of toxoplasmosis in livestock and poultry in Ningxia Chin J Vet Sci Technol 1987 6 22 24 In Chinese

[B18] Bian QH Bai JY Su DS Liu BS Liu XF Seroepidemiological survey of toxoplasmosis in humans and livestock in Shanxi, Gansu, Ningxia and Qinghai Chin J Vet Sci Technol 1988 1 17 19 In Chinese

[B19] Feng XR Wang SR Serological investigation report of *Toxoplasma gondii* among livestock in Henan Province J China Agricultural University 1998 2 172 173 In Chinese

[B20] Zhang WD Preliminary Investigation of *Toxoplasma gondii* antibodies in livestock in South China Chin J Zoonoses 1986 2 55 In Chinese

[B21] Alanazi AD Alyousif MS Prevalence of antibodies to *Toxoplasma gondii* in horses in Riyadh Province, Saudi Arabia J Parasitol 2011 5 943 945 21506811 10.1645/GE-2677.1

[B22] Hajialilo E Ziaali N Harandi MF Saraei M Hajialilo M Prevanlence of anti-Toxoplasma gondii antibodies in sport horses from Qazvin, Iran Trop Anim Health Prod 2010 42 1321 1322 10.1007/s11250-010-9576-4 20383793

[B23] Haridy FM Shoukry NM Hassan AA Morsy TA ELISA-seroprevalence of *Toxoplasma gondii* in draught horses in Greater Cairo, Egypt J Egypt Soc Parasitol 2009 3 821 826 20120748

[B24] Boughattas S Bergaoui R Essid R Aoun K Bouratbine A Seroprevalence of *Toxoplasma gondii* infection among horses in Tunisia Parasit Vectors 2011 4 218 10.1186/1756-3305-4-218 22107730 PMC3253060

[B25] Evers F Garcia JL Navarro IT Zulpo DL Nino BD Ewald MP Pagliari S Almeida JC Freire RL Diagnosis and isolation of *Toxoplasma gondii* in horses from Brazilian slaughterhouses Rev Bras Parasitol Vet 2013 doi.org/10.1590 10.1590/s1984-29612013005000009 23538498

[B26] García-Bocanegra I Cabezón O Arenas-Montes A Carbonero A Dubey JP Perea A Almería S:Seroprevalence of Toxoplasma gondii in equids from southern Spain Parasitol Int 2012 3 421 424 22366344 10.1016/j.parint.2012.02.003

[B27] Karatepe B Babür C Karatepe M Kılıc S Seroprevalence of toxoplasmosis in horses in Niğde Province of Turkey Trop Anim Health Prod 2010 42 385 389 10.1007/s11250-009-9430-8 19701805

[B28] Alvarado-Esquivel C Rodríguez-Peña S Villena I Dubey JP Seroprevalence of *Toxoplasma gondii* infection in domestic horses in Durango State, Mexico J Parasitol 2012 5 944 945 22559329 10.1645/GE-3174.1

[B29] Gupta GD Lakritz J Kim JH Kim DY Kim JK Marsh AE Seroprevalence of *Neospora*, *Toxoplasma gondii* and *Sarcocystis neurona* antibodies in horses from Jeju island, South Korea Vet Parasitol 2002 106 193 201 10.1016/S0304-4017(02)00064-X 12062508

[B30] Jakubek EB Lunde NA Uggla A Seroprevalence of *Toxoplasma gondii* and *Neospora* sp. Infections in Swedish horses Vet Parasitol 2006 138 194 199 10.1016/j.vetpar.2006.02.002 16517077

[B31] Dubey JP Thulliez P Weigel RM Andrews CD Lind P Powell EC Sensitivity and specificity of various serologic tests for detection of *Toxoplasma gondii* infection in naturally infected sows Am J Vet Res 1995 56 1030 1036 8533974

[B32] Zou FC Sun XT Li B Xie YJ Zhao GH Duan G Zhu XQ Seroprevalence of *Toxoplasma gondii* in pigs in Southwestern China Parasitol Int 2009 58 306 307 10.1016/j.parint.2009.06.002 19523533

[B33] Wang CR Qiu JH Gao JF Liu LM Wang C Liu Q Yan C Zhu XQ Seroprevalence of *Toxoplasma gondii* infection in sheep and goats in northeastern China Small Ruminant Res 2011 97 130 133 10.1016/j.smallrumres.2011.02.009

[B34] Qiu JH Wang CR Zhang X Sheng ZH Chang QC Zhao Q Wu SM Zou FC Zhu XQ Seroprevalence of *Toxoplasma gondii* in cattle and dairy cows in Northeast China Foodborne Pathogen Dis 2012 9 579 582 10.1089/fpd.2011.1104 22545962

[B35] Chang QC Zheng X Qiu JH Wang CR Zhu XQ Seroprevalence of *Toxoplasma gondii* infection in fattening pigs in Northeast China J Parasitol 2013 99 544 545 10.1645/12-102.1 23116060

